# The predictive value of microRNA-126 in relation to first line treatment with capecitabine and oxaliplatin in patients with metastatic colorectal cancer

**DOI:** 10.1186/1471-2407-12-83

**Published:** 2012-03-08

**Authors:** Torben Frøstrup Hansen, Flemming Brandt Sørensen, Jan Lindebjerg, Anders Jakobsen

**Affiliations:** 1 Department of Oncology, Vejle Hospital, Kabbeltoft 25, 7100 Vejle, Denmark; 2 Department of Clinical Pathology, Vejle Hospital, Kabbeltoft 25, 7100 Vejle, Denmark; 3 Danish Colorectal Cancer Group South, Vejle Hospital, Kabbeltoft 25, 7100 Vejle, Denmark

**Keywords:** Angiogenesis, Chemotherapy, Colorectal neoplasms, microRNAs, Predictive biomarkers

## Abstract

**Background:**

MicroRNA-126 is the only microRNA (miRNA) known to be endothelial cell-specific influencing angiogenesis in several ways. The aim of the present study was to analyse the possible predictive value of miRNA-126 in relation to first line capecitabine and oxaliplatin (XELOX) in patients with metastatic colorectal cancer (mCRC).

**Methods:**

The study included 89 patients with mCRC. *In situ *hybridization (ISH) was performed to detect miRNA-126 in formalin-fixed paraffin embedded tissue from primary tumours. The expression of miRNA-126, area per image (μm^2^), was measured using image analysis. Clinical response was evaluated according to RECIST. Progression free survival (PFS) was compared using the Kaplan-Meier method and the log rank test. Tumours were classified as low or high miRNA-126 expressing tumours using the median value from the patients with response as cut-off.

**Results:**

The median miRNA-126 expression level was significantly higher in patients responding to XELOX, 3629 μm^2 ^(95% CI, 2566-4846), compared to the patients not responding, 1670 μm^2 ^(95% CI, 1436-2041), *p *< 0.0001. The positive predictive value was 90%, and the negative predictive value was 71%. The median PFS of patients with high expressing tumours was 11.5 months (95% CI, 9.0-12.7 months) compared to 6.0 months (95% CI, 4.8-6.9 months) for patients with low expressing tumours, *p *< 0.0001.

**Conclusions:**

Angiogenesis quantified by ISH of miRNA-126 was related to response to first line XELOX in patients with mCRC, translating to a significant difference in PFS. The predictive value of miRNA-126 remains to be further elucidated in prospective studies.

## Background

In the recent years, a rapidly growing number of treatment modalities have become available for the treatment of patients with metastatic colorectal cancer (mCRC). It is therefore more important than ever to identify the right patients for the right treatment. Combination chemotherapy constitutes the backbone of mCRC treatment and combined with anti-angiogenetic therapy this is a common first line choice.

In the search for potential biomarkers, angiogenesis, the formation of new blood vessels from pre-existing vessels, has been addressed for obvious reasons, including the introduction of anti-angiogenetic treatment. Furthermore, the important role of angiogenesis in the growth and dissemination of malignant tumours [1-3] argues for testing biomarkers related to the neo-formation of the vasculature in malignancies.

MicroRNAs (miRNAs) are a group of small, non-coding RNAs that regulate several biological functions, and increasing evidence supports a pivotal role in the regulation of pathological processes as well [4,5]. The number of reports on miRNAs and their involvement in CRC pathogenesis is rapidly growing [6] and a relationship between miRNA expression patterns and response to cytotoxic treatment has also been demonstrated [7-10]. MicroRNA-126 [GenBank: NR_029695] is so far the only identified endothelial cell (EC)-specific miRNA [11] and its important role in regulating angiogenesis has been demonstrated in a few studies [12-14]. High levels of miRNA-126 expression have been correlated with increased vascular endothelial growth factor A (VEGF-A) mediated signalling in ECs and a higher blood vessel integrity [12,14,15]. Thus, current evidence seems to support a close relationship between miRNA-126 and physiological angiogenesis, but the mechanism of the regulation under different normal and malignant conditions remains to be elucidated. MicroRNA-126 is often referred to as a tumour suppressor in primary tumours and cancer cell lines [16-20]. Thus, inhibition of cancer cell-growth has been demonstrated after restoration of miRNA-126 levels [17,19,21]. Based on the current knowledge about angiogenesis, blood vessel structure, interstitial tumour pressure and their influence on the delivery of chemotherapy to the tumour cells [22-25], one could hypothesise that miRNA-126 would likewise harbour predictive information.

The aim of this study was to analyse the possible predictive value of miRNA-126 in patients with mCRC in relation to first line treatment with capecitabine and oxaliplatin (XELOX).

## Methods

### Study population, sampling and treatment regimen

The study included 89 patients with mCRC histologically verified at the Department of Clinical Pathology, Vejle Hospital, Denmark (Table 1). First line chemotherapy was initiated in the period from May 2004 to December 2009. Pre-treatment examination consisted of standard hematologic parameters and a CT scan of the chest and abdomen or a chest x-ray and ultrasound of the abdomen. All patients received a minimum of 3 cycles of XELOX. Formalin fixed paraffin embedded (FFPE) tumour tissue originating from the primary tumour was available from all patients. Patients having received preoperative chemoradiation of rectal cancer were not included. All patients meeting these criteria were offered inclusion in the study.

**Table 1:** Patient characteristics at time of diagnosis

	Number (%)	miRNA-126 expression level (μm^2^)	
	(n = 89)	Median (95% CI)	p - value
Sex			

Male	45 (51)	2533 (1690-3285)	0.84

Female	44 (49)	2354 (1650-3163)	

Age (years)			

Mean (SD)	63.2 (8.2)		

Range	24 - 80		

> mean	49 (55)	2088 (1556-3223)	0.56

< mean	40 (45)	2531 (1896-3355)	

ECOG Performance status			

0	37 (42)	2041 (1551-3163)	0.40

1-2	52 (58)	2535 (1708-3489)	

Localization			

Rectum	18 (20)	3038 (1690-4030)	0.37

Colon	71 (80)	2141 (1650-2638)	

Left colon	47 (66)	2141 (1589-2818)	0.99

Right colon	24 (34)	2232 (1452-3355)	

MSI status*			

MSI	8 (11)	2193 (1374-5421)	0.61

MSS	62 (89)	2355 (1705-2818)	

Metastatic sites*			

1	27 (31)	3575 (3163-4521)	**< 0.0001**

≥ 2	59 (69)	1857 (151-2376)	

*CI *confidence interval, *SD *standard deviation, *ECOG *Eastern Cooperative Oncology Group, *MSI *microsatellite instability, *MSS *, microsatellite stable

*MSI status was not assessed in all patients

**A reliable estimate of the number of metastatic sites was not possible in 3 patients

Two patients had a previous diagnosis of breast cancer, but had no sign of relapse at the time of diagnosis of their CRC, and their survival data were consequently left uncensored. No patients were diagnosed with a new malignancy after their CRC diagnosis. Progression free survival data were censored in 10 cases. Six patients initiated a second-line treatment before progression and data were censored from the first day of their new treatment. Two patients underwent radio frequency ablation, and two patients had surgical resection of their liver metastases and their data were censored from the day of the intervention.

Immediately after surgery the removed bowel segment was brought to the Department of Clinical Pathology and a pathologist sampled tissue from the tumour. Samples intended for later *in situ *hybridization (ISH) analyses followed routine fixation and paraffin embedding. Postoperatively, the tumours were histologically classified and staged according to the pTNM system. Information regarding patient characteristics, relapse status and survival were based on patient records, pathology reports, and a national registry based on central person registration numbers.

All patients received the same treatment (XELOX), which consisted of a 2-hour intravenous infusion of oxaliplatin 130 mg/m^2 ^on day 1 followed by oral capecitabine 1000 mg/m^2 ^twice daily on days 1 through 14 (28 doses) of a 21-days cycle. Treatment was continued until disease progression or unacceptable toxicity.

The study was approved by the Regional Scientific Ethical Committee for Southern Denmark according to Danish law, J.nr. S-VF-20040047, and informed consent was obtained from all patients enrolled in the study.

### Evaluation and tumour response criteria

Treatment response, according to RECIST (version 1.0), was assessed every 9 weeks with clinical and radiologic examination using CT scan of the chest and abdomen (86 patients) or a chest x-ray and ultrasound of the abdomen (3 patients). Responding patients were classified as having either complete response (CR) or partial response (PR). Patients with stable disease (SD) or progressive disease (PD) were defined as non-responders. All CT scans were evaluated by the same investigator (TFH).

### LNA probe, in situ hybridization, image analysis and quantification

The entire ISH procedure and the subsequent quantification was performed at Exiqon, Vedbaek, Denmark and followed previously described procedures [26,27].

In brief, the ISH analysis was carried out on 6-μm thick FFPE tissue sections containing CRC tissue, using a double digoxigenin (DIG)-labelled mercury Locked Nucleic Acid (LNA) probe (LNA™ microRNA detection probe, Exiqon, Vedbaek, Denmark) specific for human miRNA-126. Tissue sectioning was performed in an RNase-free environment.

An initial assay optimization was performed on nine sections from five CRC samples in order to determine the optimal probe concentration and hybridization temperature. The ISH assay involved a protein-K treatment (15 μg/ml) followed by a hybridization step, using the LNA probe at 57°C and stringent washes. The DIG-labelled probe was detected with alkaline phosphatase conjugated sheep anti-DIG Fab fragments followed by NBT-BCIP chromogenic staining and nuclear fast red staining.

**Figure 1:**
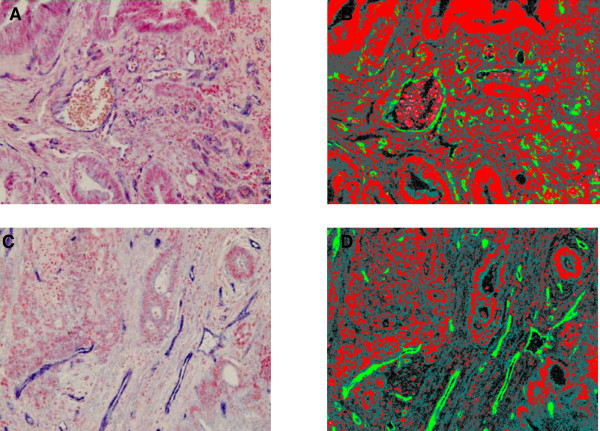
**In situ hybridization (ISH) visualising the expression of miRNA-126 in tumour vasculature. A **represents an image from a patient with colon cancer in which the mean ISH signal (the blue signal) from all the analysed images resulted in an area of 4280 μm^2^. **B **the pixel classifier corresponding to image A. **C **also represents an image from a patient with colon cancer with a mean ISH signal of 2736 μm^2^. **D **the pixel classifier corresponding to image C.

For image acquisition up to 25 random, systematically positioned images were collected from the tumour slide. Initially, the whole tumour area was encircled to avoid ulcerating, necrotic and normal tissue. Images containing distorted tissue and staining artefacts were deleted and only images with cancer cells and tumour stroma were included. A corresponding pixel classifier included the following colours for the identification of the stained histological structures. Intense blue (the ISH signal), weak blue (background ISH signal), red (nuclear stain), red spot (mucin), black spot (artefact), unstained (no-tissue and erythrocytes). The area of the ISH signal (μm^2 ^per image), was used as the parameter reflecting miRNA-126 expression levels, and the mean score from the sampled images (at × 20 magnification) was used for each patient. Figure 1 provides examples of the underlying pixel classifier. The entire ISH procedure was performed by a staff unaware of the clinical parameters.

### Statistical analysis

Fisher's exact test was used for two-group comparisons. Median values were compared using the Wilcoxon rank sum test. Progression free survival (PFS) was defined as the time from start of treatment until the first documented tumour progression or death. Overall survival (OS) was defined as the time from start of treatment until death. All survival data were complete. Survival curves were illustrated according to the Kaplan-Meier method and the logrank test was used to test for differences between the groups. All statistical calculations were carried out using the NCSS statistical software (NCSS Statistical Software, Kaysville, UT 84037, USA, version 2007). *P *values < 0.05 were considered significant, and all tests were two-sided.

## Results

The miRNA-126 ISH resulted in a strong signal in various vessels throughout the tumour in all the samples (Figure 1). No apparent unspecific signals were detected from the extracellular matrix, but background signal was detected in a few samples caused by tumour sections being too thick.

### Patient characteristics

The patient characteristics along with the miRNA-126 expression levels in the primary tumours are shown in Table 1. A significant relationship was demonstrated between the number of metastatic sites and the median miRNA-126 expression level. The presence of two or more metastatic sites was related to lower miRNA-126 expression levels compared to the expression levels in patients with metastases limited to one site.

### The predictive value of miRNA-126 in relation to XELOX

Eighty-three patients were included in the analyses of the predictive value of miRNA-126 in relation to first line XELOX in mCRC. Five patients were excluded due to the fact that XELOX was given as second or third line treatment, and one patient was excluded because of insufficient response evaluation according to the RECIST criteria.

**Figure 2:**
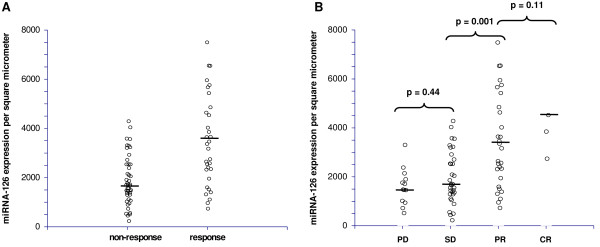
**Relationship between miRNA-126 and response**. (**A**) Distribution of miRNA-126 expression levels according to response or non-response to first line XELOX in patients with mCRC, black bars represent the medians, n = 83. The difference between the medians in the two groups was significant, *p *= 0.000005. (**B**) Illustration of the same distribution divided into the four response groups (PD; progressive disease, SD; stable disease, PR; partial response and CR; complete response). A significant difference between the median miRNA-126 expression levels in the patients achieving SD compared to PR was observed, *p *= 0.001. The miRNA-126 expression levels from five patients (outliers, levels ranging from 16775-40284) are not shown in the figures for graphical reasons only, but were included in the statistics. Two of these patients achieved PR and the remaining three CR.

The distribution of miRNA-126 expression levels according to response is shown in Figure 2. The median miRNA expression level was significantly higher in the responding patients, 3629 (95% CI 2566-4846) compared to the non-responding patients, 1670 (95% CI 1436-2041), *p *= 5 × 10^-6^. The largest difference in median miRNA-126 expression levels was demonstrated when comparing patients achieving SD to patients achieving PR.

In order to assess the predictive value of the miRNA-126 ISH analysis in the context of predicting response to first line XELOX treatment, patients were divided into two groups. The median value (3629 μm^2^) from the response group was used as cut-off. Applying this strategy, the predictive value of a positive test ( > 3629 μm^2^) was 19/21 = 90%, and of a negative test ( < 3629 μm^2^) was 44/62 = 71%.

**Figure 3:**
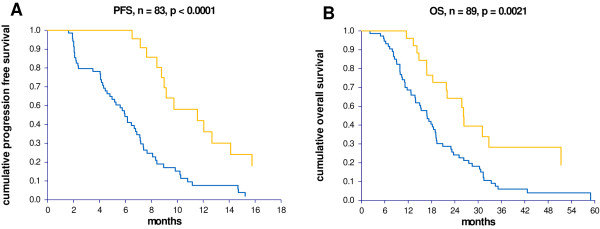
**Kaplan-Meier survival curves according to miRNA-126 expression levels**. (**A**) Illustrates the progression free survival (PFS) curves and (**B**) the overall survival (OS) curves. The yellow lines represent patients with high miRNA-126 expression levels and the blue lines patients with low miRNA-126 expression. The difference between the curves was significant in the PFS comparison, *p *< 0.0001, as well as in the OS comparison, *p *= 0.0021.

The same division of the patients was used in the subsequent PFS analysis (Figure 3A). The difference in response rates between the two groups reflected a significant difference in PFS, *p *< 0.0001. The median PFS for patients with high miRNA-126 expression levels was 11.5 months (95% CI 9.0-12.7 months) compared to 6.0 months (95% CI 4.8-6.9 months) for patients with low expression levels. The difference in PFS translated into a significant difference in OS as well, *p *= 0.0021 (Figure 3B). The median OS in the group with high miRNA-126 expression was 26.2 months (95% CI 21.8-32.8 months) compared to 16.8 months (95% CI 13.8-19.1 months) in the group with low miRNA-126 expression.

The median overall survival for the entire cohort was 18.5 months (95% CI 16.7-21.8 months).

A subgroup analysis comparing patients with colon cancer and rectum cancer did not change any of the presented results.

## Discussion

An increasing number of pre-clinical studies have demonstrated a pivotal role of miRA-126 in regulating angiogenesis, a process that has also been related to the delivery and the efficacy of chemotherapy. Little is known, though, about the in vivo localisation of miRNA-126 in human CRC tissue samples, but the present results support the previous studies indicating specificity towards ECs. In the present study we analysed the possible predictive value of miRNA-126 in relation to first line XELOX therapy in patients with mCRC, using a quantitative analysis of the miRNA-126 expression level based on ISH analyses of tumour sections from the primary tumour. This method has recently demonstrated its reliability in predicting short disease-free survival in stage II colon cancer patients [26].

A significant relationship between miRNA-126 expression levels and number of metastatic sites was demonstrated. The miRNA-126 expression level was significantly lower in patients with two or more metastatic sites compared to the patients with disease limited to only one metastatic location. This could indicate that the molecular genetic features of the tumour cells originating from the primary tumours with low levels of miRNA-126 are related to the ease by which these circulating tumour cells invade tissues and gives rise to distant metastases. This seems to apply rather well with the conception of miRNA-126 functioning as a tumour suppressor.

In the present study a significant relationship between miRNA-126 expression levels in the primary tumour and response to first line XELOX treatment was demonstrated for patients with mCRC. Previous studies have also reported on predictive value of markers related to angiogenesis in patients with mCRC [28,29]. The present marker, miRNA-126, is related to vessel integrity [15], and correlations between vessel structure and response to chemotherapy have previously been demonstrated [24,25] supporting the plausibility of the present results. Furthermore, a study by Zhou *et al. *[9] reported on changes of miRNA expression profiles in colon cancer cell lines following exposure to XELOX. Low expression levels of miRNA-126 may therefore represent tumour vessels with lower integrity and the expected increase in the interstitial tumour pressure that follows may explain the lower response rates seen in these patients.

Dividing the patients by cut-off method, i.e. median miRNA-126 expression level, demonstrated that the observed relationship with response rates was also translated into a corresponding difference in PFS. A difference in OS was also demonstrated, when comparing patients with low and high miRNA-126 expression levels. The relationship with OS may indicate that miRNA-126 has a prognostic importance in addition to the predictive value. We did not find it indicated to perform a multivariate survival analysis adjusting for parameters of prognostic importance in stage I through III disease in this cohort of patients with stage IV disease. In the study by Díaz *et al. *no prognostic value of miRNA-126 was demonstrated in patients with stage I to III colon cancer [30]. The disease stage of the included patients constitutes one of the major differences between their study and ours, which included patients with stage IV disease only. Schepeler *et al. *showed that miRNA-126 was up-regulated in patients with subsequent recurrence compared to patients with no recurrence in a cohort of 37 patients operated for stage II colon cancer with microsatellite stable tumours only [31]. Differences in methods, sample size, and stage of the disease again represent the most obvious explanations of these discrepancies, but the results from the three studies indicate that the possible prognostic value of miRNA-126 remains to be further clarified. If indeed, assessment of miRNA-126 expression in the primary tumour from patients with CRC harbours prognostic value, one would expect a better prognosis for patients with higher expression levels of this miRNA if functioning as a tumour suppressor.

The present study has the standard limitations of a retrospective study; a rather limited sample size and results obtained form a single institution. Having said that it also represents a well argued hypothesis, a reliable method, and results of clinical relevance in line with the preclinical literature.

An alternative technique to quantify angiogenesis is presented based on ISH of miRNA-126 in primary tumour tissue and using a specific LNA based probe and new quantitative systematic image analyses. This approach resulted in an estimate related to disease characteristics, the response to chemotherapy, and the prognosis of patients with mCRC. The new application of the ISH method presented here indicated that miRNA-126 may be an important predictive marker to chemotherapy applied in the clinical setting, but the results call for validation in a prospective trial. Furthermore, it shall be interesting to analyse the possible predictive value of miRNA-126 in patients with mCRC treated with chemotherapy combined with anti-VEGF-A.

## Abbreviations

mCRC: metastatic colorectal cancer; miRNA: microRNA; CRC: colorectal cancer; EC: endothelial cell; VEGF-A: vascular endothelial growth factor A; XELOX: capecitabine and oxaliplatin; CT: computer tomografi; FFPE: formalin fixed paraffin embedded; ISH: in situ hybridization; RECIST: response evaluation criteria in solid tumors; CR: complete response; PR: partial response; SD: stable disease; PD: progressive disease; DIG: double digoxigenin; LNA: locked nucleic acid; PFS: progression free survival; OS: overall survival; CI: confidence interval.

## Competing interests

The authors declare that they have no competing interests.

## Authors' contributions

All authors contributed equally to the conception and design. TFH performed the data evaluation, interpretation and manuscript drafting. FBS, JL and AJ performed critical revisions. All authors read and approved the final manuscript.

## Pre-publication history

The pre-publication history for this paper can be accessed here:

http://www.biomedcentral.com/1471-2407/12/83/prepub
